# Supplementary material for: KBeagle: An Adaptive Strategy and Tool for Improving Imputation Accuracy and Computation Time
Source: Int J Mol Sci. 2025 Jun 18;26(12):5797. doi: 10.3390/ijms26125797 (PMC12192696; doi:10.3390/ijms26125797)
Supplement: Supplementary file 1 [file ijms-26-05797-s001.zip › ijms-3657592-supplementary.pdf]

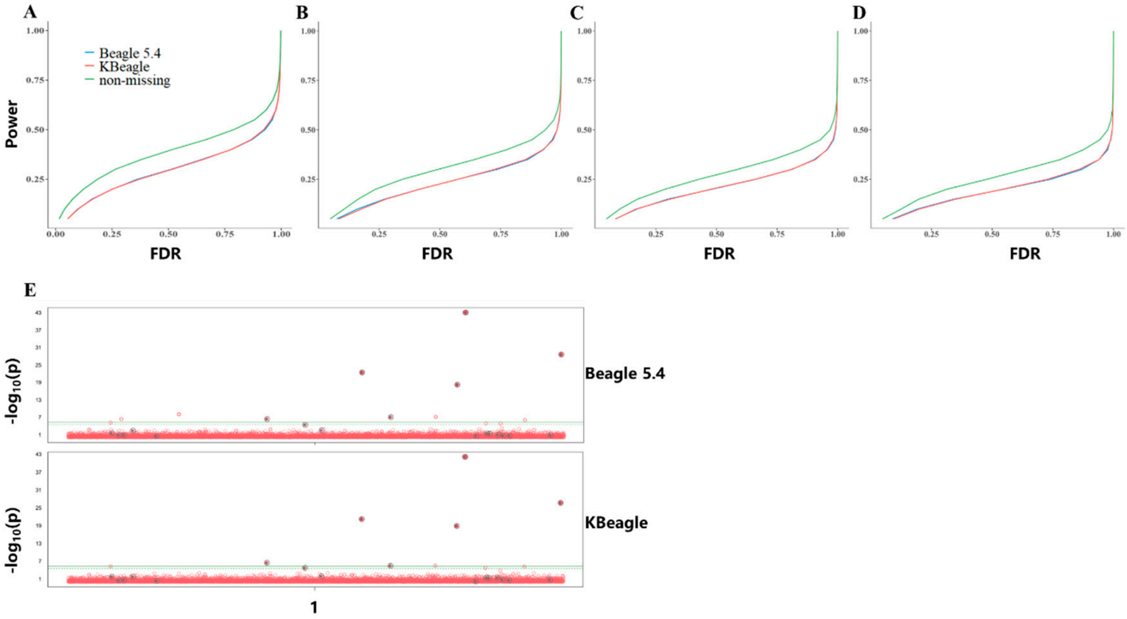

Figure S1 The results of GWAS with Ashidan yaks. (A-D) The plots showed the power of three types of genotype datasets including ND, as well as BID and KID under different FDR of Ashidan yaks. (E) The plots indicated the distribution of SNP sites of BID (above) and KID (below) from Ashidan yaks.

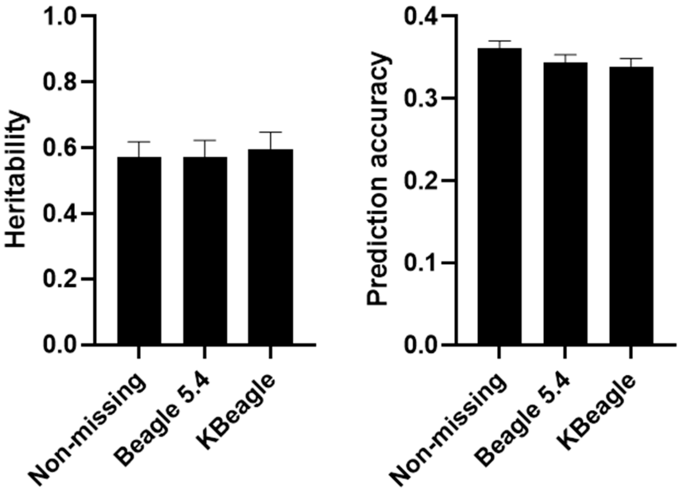

Figure S2 Genomic prediction of Ashidan yaks under three types of genotype datasets. In genomic prediction, the study primarily compared the estimated heritability (A) and prediction accuracy (B) of ND, as well as BID and KID with the same missing rate (0.1) under 10K.

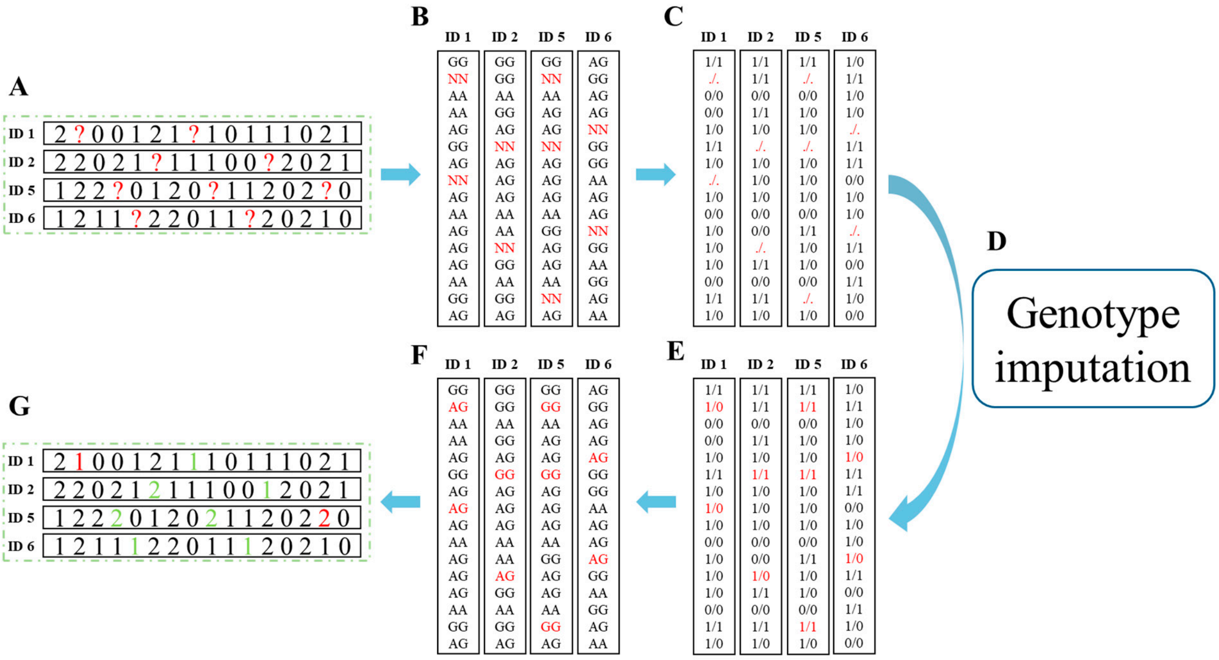

Figure S3 Adapter of converting file format. The Adapter progress contained 7 steps: (A)Numeric data Inputting; (B)Hapmap data; (C)VCF data; (D)Imputing; (E)VCF data; (F)Hapmap data; (G) Numeric data outputting.
